
JOURNAL INFORMATION
==============================
NLM Title Abbreviation: Chic Med J
NLM Journal ID: 0027576

The Chicago Medical Journal

Publisher: Biomedical Journal Digitization Project

ARTICLE INFORMATION
==============================
PMCID: PMC9721565
PMID: 37411068
Article version: 1
Subjects: Editorial

Hymenial

Print publication date: 1858 Jan
Volume: 15
Issue: 1
First page: 50
Last page: 50
License: This work is free of known copyright restrictions. For more information, please see PMC Back Issue Digitization.
License URL: https://creativecommons.org/publicdomain/mark/1.0/

==============================

---

Interesting communications have been received from Drs. J. N. Graham, J. W. Crooks, Charles Brackett, J. P. DeBruler, C. Goodbrake, P. A. Allaire, and E. Read. They shall appear as fast as our space will permit; and the writers have our warmest thanks for their kindness. A short, but interesting article, came post marked Jacksonville. If the writer will send us his name, we will take great pleasure in publishing it with the article.

---

#### HOMŒOPATHY AND THE CHICAGO CITY HOSPITAL.

We see that several of our exchanges are representing this city as the only one in the country containing a public hospital under homœopathic charge. We would inform all such that our city hospital is not yet under the charge of any medical faculty. It has never been opened for the reception of patients, and we do not know when it will be.

---

#### HYMENIAL.

Married, in Orange, New Jersey, Nov. 28th 1857, by the Rev. F. A. Adams, D.D., J. B. Moffett, M.D., of Mineral Point, Wis., to Miss H. A. Larned, of Watertown, N. Y.

---

### TO THE MEDICAL PROFESSION.

**The subscribers would call the attention of physicians to the annexed list of Fluid Extracts, which we have been induced to prepare, from the difficulty of obtaining such preparations of a reliable character, and to obviate the great inconvenience of being dependent on distant manufacturers for articles of every day use by physicians.**
